# Temporal trends in patients undergoing artificial urinary sphincter implantation: explantation outcomes and early failure patterns in a tertiary reconstructive referral center

**DOI:** 10.1007/s00345-026-06899-9

**Published:** 2026-11-13

**Authors:** Navid Roessler, Malte W. Vetterlein, Jakob Klemm, Max C. Wagner, Roland Dahlem, Margit Fisch, Tim A. Ludwig

**Affiliations:** Department of Urology, University Medical Center Hamburg-Eppendorf, Martinistraße 52, 20246 Hamburg, Germany

**Keywords:** Artificial urinary sphincter, Stress urinary incontinence, Explantation, Risk factors, Temporal trends, AUS

## Abstract

**Background:**

Baseline characteristics and perioperative risk profiles in patients undergoing artificial urinary sphincter (AUS) implantation have changed over time, potentially influencing clinical outcomes. This study assessed temporal trends in patient characteristics and explantation outcomes to inform preoperative counselling and risk stratification.

**Materials and methods:**

This retrospective study included male patients undergoing AUS implantation between 2009 and 2025, stratified into four implantation periods. Explantation-free survival was analyzed using the Kaplan-Meier method. Risk factors for explantation were evaluated using multivariable Cox proportional hazards regression models.

**Results:**

A total of 491 patients were included (2009–2012: *N* = 177; 2013–2017: *N* = 159; 2018–2022: *N* = 107; 2023–2025: *N* = 48), with a median follow-up of 54 months (IQR 14–116). Baseline characteristics were largely comparable, with a relative increase in prior radiotherapy from 35% to 42% over time. Explantation rates were 36%, 27%, 22%, and 17%, respectively, predominantly due to erosion and infection. In multivariable analysis, prior radiotherapy (HR 1.88, 95% CI 1.30–2.70), prior BPH surgery (HR 1.75, 95% CI 1.10–2.77), and prior bulbar urethroplasty (HR 2.10, 95% CI 1.12–3.93) were independent predictors of explantation. Kaplan-Meier analysis showed no significant differences in explantation-free survival between periods (*p* = 0.8), although a numerical increase in early explantations within 24 months was observed (7.9% to 13%). In a supplementary analysis focusing on early explantation, prior radiotherapy (HR 2.46, 95% CI 1.38–4.39) and prior urethral surgery (HR 2.50, 95% CI 1.31–4.77) were independently associated with increased risk. Later implantation periods showed an increase in early explantation risk; however, this did not reach statistical significance. The study is limited by its retrospective single-center design.

**Conclusion:**

The AUS remains a durable and effective treatment option for male incontinence, with stable outcomes across implantation periods. Careful preoperative counselling and structured follow-up remain essential, particularly in high-risk patients.

**Supplementary Information:**

The online version contains supplementary material available at https://doi.org/10.1007/s00345-026-06899-9.

## Introduction

The artificial urinary sphincter (AUS) represents the cornerstone of surgical management for moderate-to-severe male stress urinary incontinence (SUI) [1]. Over the past decades, its surgical principle and role as the gold standard therapy have remained unchanged, consistently demonstrating high continence rates alongside meaningful improvements in patient-reported quality of life [2–4].

Concurrently, the patient population undergoing AUS implantation may have changed substantially over recent decades, with contemporary patients presenting a higher comorbidity burden and more complex urological treatment histories, reflecting demographic shifts and prior interventions such as radiotherapy and urethral surgery [5]. Current candidates for AUS implantation may therefore represent a more heterogeneous and higher-risk cohort compared to earlier populations, a shift that has important implications for risk stratification and clinical outcomes, underscoring the relevance of the concept of the fragile urethra in recent urological practice [6].

Although the underlying device principles and surgical concepts have remained largely unchanged, increasing patient complexity may influence the overall treatment course. These developments do not question the role of the AUS itself but rather highlight the need to better understand how changing patient characteristics interact with outcomes in male incontinence surgery. In this context, particular attention should be paid to optimizing preoperative counselling, aligning patient expectations, and adapting follow-up strategies to reflect the growing complexity of these patients. While individual risk factors for AUS explantation have been well characterized in large cross-sectional and multicentre cohorts [7], it remains less well established how these known risk profiles translate into changing failure patterns, particularly regarding the timing of device failure, within a single institution over an extended observation period.

With this study, we aimed to provide a trend-oriented perspective on AUS implantation from a high-volume reconstructive referral center by characterizing patients across defined time periods and to assess whether changing patient characteristics translate into clinically relevant implications for device performance and outcomes, thereby informing standardized and individualized perioperative counselling as well as more uniform follow-up strategies in patients undergoing AUS implantation.

## Methods

The study protocol was approved by the Institutional Review Board (2021–100628-BO-ff).

### Study population

This study included all male patients undergoing AUS implantation at our institution between beginning of January 2009 and end of January 2025 who presented without an indwelling continence device at the time of evaluation, ensuring a homogeneous cohort regarding standardized perioperative management and preoperative counselling. Perioperative and follow-up morbidity data were prospectively collected from electronic medical records (Soarian Clinicals). Data extraction from electronic medical records was performed systematically and transferred into a dedicated REDCap (Research Electronic Data Capture) database. Follow-up was conducted by identifying patients through the institutional database and contacting them via structured telephone interviews. In addition, consenting patients received an email containing a REDCap survey link to complete standardized questionnaires at a single follow-up time point. All follow-up data were subsequently integrated into the same database. Follow-up was defined as the interval from AUS implantation to the last contact, either in person, by telephone, or at the time of device explantation.

### Clinical and surgical characteristics

Preoperatively, patients underwent a standardized clinical assessment during which comorbidities and relevant risk factors were systematically documented. Preoperative data included patient demographics (e.g., age, body mass index, smoking status) and comorbidities (e.g., hypertension, hyperlipidaemia, diabetes mellitus). The overall comorbidity burden was additionally quantified using the age-adjusted Charlson Comorbidity Index (CCI) [8]. In addition, detailed clinical history was recorded, including the etiology of SUI, prior pelvic radiotherapy, benign prostatic hyperplasia (BPH) surgery, previous incontinence-related procedures such as urethral bulking, male sling or AUS implantation, as well as prior urethral instrumentation and reconstructive procedures (e.g., direct vision internal urethrotomy [DVIU], bulbar urethroplasty, and complex urethral reconstructive procedures). All eligible patients presented with moderate-to-severe SUI confirmed by preoperative urodynamic assessment. Patients with bladder capacity < 300 mL or detrusor overactivity were not considered candidates for AUS implantation and were excluded from the analysis.

All patients received perioperative intravenous antibiotic prophylaxis (cefuroxime and gentamicin) according to the institutional standard protocol. Intraoperative data comprised the American Society of Anesthesiologists (ASA) physical status and implanted AUS cuff type. AUS implantation was performed following a standardized surgical protocol, as previously described [9]. Patients without urethral risk factors received a proximal bulbar single cuff, whereas those with risk factors, such as prior urethroplasty, pelvic radiotherapy, previous incontinence surgery, or preoperative evidence of urethral damage assessed by cystoscopy or urethrography, were treated with a distal double cuff. In cases of prior double cuff explantation or pronounced urethral fragility, a transcorporal cuff was implanted as a salvage option [10].

The AUS system remained deactivated postoperatively, and a 12 F Foley catheter was placed and left in situ for three days. Follow-up followed institutional protocols, with all patients returning six weeks post-implantation for device activation and clinical evaluation. Additional follow-ups were scheduled at six and 24 months, and every two years thereafter.

### Statistical analysis

All statistical analyses were performed using R software (R Foundation for Statistical Computing, Vienna, Austria). A two-sided p-value < 0.05 was considered statistically significant. Continuous variables are presented as median and interquartile range (IQR), while categorical variables are reported as absolute numbers and percentages. Baseline characteristics were stratified by predefined implantation periods (2009–2012, 2013–2017, 2018–2022, and 2023–2025) to evaluate temporal trends in patient characteristics and surgical practice. Categorical variables were compared using Chi-square or Fisher’s exact test as appropriate, while continuous variables were analyzed using the Kruskal-Wallis test.

The primary endpoint was explantation-free survival, defined as the time from AUS implantation to explantation of any device component or the entire device for any reason, leading to loss of device function. Explantation-free survival was analyzed using the Kaplan-Meier method. Patients without explantation were censored at the date of last follow-up. To avoid bias due to insufficient follow-up in the most recent cohort, survival analyses were restricted to the first three time periods. Median follow-up time was estimated using the reverse Kaplan-Meier method. Survival curves were compared using the log-rank test, and numbers at risk were displayed at predefined time points.

Explantation rates were calculated for each cohort, and reasons for explantation were analyzed descriptively. To identify risk factors for explantation, univariable and multivariable Cox proportional hazards regression models were performed. Results are reported as hazard ratios (HRs) with 95% confidence intervals (CIs). In a supplementary analysis, early explantation within the first 24 months after AUS implantation was assessed using a multivariable Cox proportional hazards regression model. The model was adjusted for established risk factors defining a fragile urethra, including prior radiotherapy, prior urethral surgery (bulbar urethroplasty and/or complex urethral reconstructive procedures), and prior incontinence surgery (prior male sling and/or AUS implantation), as well as implantation period, given their reported association with early explantation risk [11].

## Results

After exclusion of female patients and individuals with an indwelling continence device at the time of evaluation, 491 male patients were included in the final analysis. Patients were stratified according to year of implantation into four time periods for temporal trend analysis: 2009–2012 (*N* = 177), 2013–2017 (*N* = 159), 2018–2022 (*N* = 107), and 2023–2025 (*N* = 48).

### Descriptive analysis of clinical and surgical characteristics

The demographic and clinical characteristics of the study cohort are summarized in Table 1. Median age at implantation ranged from 69 to 71 years, while median BMI varied between 27 kg/m² and 29 kg/m² across the observed time periods.

**Table 1:** Patient demographics and clinical characteristics at baseline, stratified by implantation period

Variable	2009–2012; *N* = 177	2013–2017; *N* = 159	2018–2022; *N* = 107	2023–2025; *N* = 48	*p*-value
Age at implantation (yr), median (IQR)					0.6
	70 (65–74)	71 (66–75)	70 (65–76)	69 (65–76)	
BMI (kg/m^2^), median (IQR)					0.003
	27 (25–29)	28 (25–31)	29 (25–31)	28 (25–30)	
Hypertension, N (%)					0.7
No	50 (28)	50 (31)	32 (30)	18 (38)	
Yes	127 (72)	109 (69)	75 (70)	30 (63)	
Hyperlipidaemia, N (%)					0.006
No	57 (32)	76 (48)	54 (50)	20 (42)	
Yes	120 (68)	83 (52)	53 (50)	28 (58)	
Diabetes mellitus, N (%)					0.1
No	157 (88)	128 (81)	84 (79)	43 (90)	
Dietary measures	2 (1.1)	2 (1.3)	0 (0)	0 (0)	
Oral medication	16 (9)	23 (15)	18 (17)	3 (6.2)	
Insulin-dependent	2 (1.1)	6 (3.8)	5 (4.6)	2 (4.2)	
Smoking status, N (%)					0.03
Never	151 (85)	140 (88)	77 (72)	41 (85)	
Former	11 (6.2)	7 (4.4)	10 (9.3)	1 (2.1)	
Active	15 (8.4)	12 (7.5)	20 (19)	6 (13)	
Etiology of SUI, N (%)					0.004
RP	123 (69)	121 (76)	85 (79)	38 (79)	
TUR-P	26 (15)	14 (8.8)	9 (8.4)	2 (4.2)	
HoLEP/ThuLEP	4 (2.3)	3 (1.9)	1 (0.9)	2 (4.2)	
Trauma	2 (1.1)	0 (0)	2 (1.9)	0 (0)	
Urethral surgery	16 (9)	10 (6.3)	8 (7.5)	5 (10)	
TUR-Bladder neck	0 (0)	5 (3.1)	1 (0.9)	1 (2.1)	
Other	6 (3.4)	6 (3.8)	1 (0.9)	0 (0)	
Prior radiotherapy, N (%)					0.4
No	115 (65)	101 (64)	57 (53)	28 (58)	
EBRT	52 (29)	52 (33)	43 (40)	19 (40)	
LDR-Brachy	7 (4)	5 (3.1)	7 (6.5)	1 (2.1)	
HDR-Brachy + EBRT	3 (1.7)	1 (0.6)	0 (0)	0 (0)	
Prior BPH surgery, N (%)					0.3
No	142 (80)	135 (85)	94 (88)	42 (88)	
Yes	35 (20)	24 (15)	13 (12)	6 (13)	
Prior urethral bulking, N (%)					0.2
No	169 (96)	157 (99)	104 (97)	48 (100)	
Yes	8 (4.5)	2 (1.3)	3 (2.8)	0 (0)	
Prior male sling, N (%)					0.2
No	146 (82)	134 (84)	95 (88)	45 (94)	
Yes	31 (18)	25 (16)	12 (11)	3 (6.2)	
Prior AUS, N (%)					0.071
No	165 (93)	155 (97)	101 (94)	42 (88)	
1	8 (4.5)	3 (1.9)	5 (4.6)	5 (10)	
2	4 (2.2)	0 (0)	1 (0.9)	1 (2.1)	
3	0 (0)	1 (0.6)	0 (0)	0 (0)	
Prior DVIU, N (%)					0.08
No	140 (79)	104 (65)	85 (79)	35 (73)	
1	13 (7.3)	24 (15)	7 (6.5)	5 (10)	
2	24 (14)	31 (20)	15 (14)	8 (17)	
Prior bulbar urethroplasty, N (%)					0.5
No	165 (93)	147 (92)	95 (89)	43 (90)	
Yes	12 (6.8)	12 (7.5)	12 (11)	5 (10)	
Prior complex urethral reconstruction, N (%)					0.5
No	161 (91)	150 (94)	99 (93)	43 (90)	
YV/T-Plasty	0 (0)	1 (0.6)	2 (1.9)	0 (0)	
Perineal reanastomosis (due to VUAS)	13 (7.3)	6 (3.8)	4 (3.7)	4 (8.3)	
Bulboprostatic reanastomosis (due to PFUI)	3 (1.7)	2 (1.3)	2 (1.9)	1 (2.1)	
ASA status, N (%)					0.1
1	3 (1.7)	0 (0)	0 (0)	2 (4.2)	
2	135 (76)	123 (77)	89 (83)	34 (71)	
3	37 (21)	30 (19)	17 (16)	12 (25)	
4	2 (1.1)	6 (3.8)	1 (0.9)	0 (0)	
CCI, N (%)					0.5
≤ 2	10 (5.6)	8 (5)	4 (3.7)	5 (10)	
3–5	94 (53)	93 (59)	65 (61)	22 (46)	
≥ 6	73 (41)	58 (37)	38 (36)	21 (44)	

*ASA * American Society of Anesthesiologists; *AUS * Artificial Urinary Sphincter; *BMI * Body Mass Index; *BPH * Benign Prostatic Hyperplasia; CCI  Charlson Comorbidity Index; *DVIU  *Direct Vision Internal Urethrotomy; *EBRT*  External Beam Radiotherapy; *HDR-Brachy *  High Dose Rate Brachytherapy; *HoLEP*  Holmium Laser Enucleation of the Prostate; *IQR*  nterquartile Range; *LDR-Brachy *Low Dose Rate Brachytherapy; *N*  Number; *PFUI*  Pelvic Fracture Urethral Injury; *RP *Radical Prostatectomy; *SUI *  Stress Urinary Incontinence; *ThuLEP*  Thulium Laser Enucleation of the Prostate; *TUR-Bladder neck  *Transurethral Resection of the Bladder Neck; TUR-P  Transurethral Resection of the Prostate; VUAS   Vesicourethral Anastomotic Stenosis

Percentages may not add up to 100%, as they are rounded.

The prevalence of hypertension remained consistently high, with the highest proportion observed in the earliest period (72%, *N* = 127, 2009–2012), compared to 63% (*N* = 30) in the most recent cohort. Hyperlipidaemia was most frequent in 2009–2012 (68%, *N* = 120), while lower proportions were observed in the subsequent periods, including 58% (*N* = 28) in 2023–2025. Diabetes mellitus was present in 11% (*N* = 20) of patients in 2009–2012 and in 21% (*N* = 23) in 2018–2022, whereas 10% (*N* = 5) of patients were affected in the most recent cohort. Most patients were never smokers in all periods; however, the proportion of active smokers was higher in 2018–2022 (19%, *N* = 20) compared to 8.4% (*N* = 15) in 2009–2012 and 13% (*N* = 6) in 2023–2025.

Radical prostatectomy was the most common etiology of SUI throughout all time periods, accounting for 69% (*N* = 123) of cases in 2009–2012 and 79% (*N* = 38) in 2023–2025. Other etiologies of SUI showed only minor variation across the cohorts. A history of pelvic radiotherapy was present in 35% (*N* = 62) of patients in 2009–2012 and in 42% (*N* = 20) in 2023–2025, with external beam radiotherapy representing the most common modality. Accordingly, patients without prior radiotherapy accounted for 65% (*N* = 115) in the earliest period and 58% (*N* = 28) in the most recent cohort. Prior BPH surgery was reported in 20% (*N* = 35) of patients in 2009–2012 and in 13% (*N* = 6) in 2023–2025.

The proportion of patients with previous AUS implantation was 6.7% (*N* = 12) in 2009–2012 and 12% (*N* = 6) in 2023–2025. Prior urethral instrumentation was common across all time periods, with repeated DVIU procedures (≥ 2) reported in up to 20% of patients, particularly in the intermediate cohorts. Prior bulbar urethroplasty was reported by 6.8% (*N* = 12) of patients in 2009–2012 and in 10% (*N* = 5) in the most recent cohort, while complex urethral reconstruction remained infrequent but present in all periods. Regarding perioperative risk, most patients were classified as ASA II (71–83%) or ASA III (16–25%). A high comorbidity burden (CCI ≥ 6) was observed in a considerable proportion of patients across all time periods (36–44%). Distal double-cuff placement was the most frequently used cuff positioning across all time periods, reflecting the high prevalence of urethral risk factors, followed by bulbar cuff placement (Supplementary File 1).

### Explantation outcomes and Kaplan–Meier analysis

Within a median follow-up of 54 months (IQR 14–116), explantation occurred in 36% of patients (*N* = 63) in 2009–2012, 27% (*N* = 43) in 2013–2017, 22% (*N* = 24) in 2018–2022, and 17% (*N* = 8) in 2023–2025. Urethral erosion was the most common reason for explantation throughout all time periods, accounting for 32% (*N* = 20) in 2009–2012, 30% (*N* = 13) in 2013–2017, 25% (*N* = 6) in 2018–2022, and 25% (*N* = 2) in 2023–2025. Infection-related complications were observed in 5% (*N* = 3), 19% (*N* = 8), 17% (*N* = 4), and 25% (*N* = 2), respectively. Combined erosion with infection accounted for 16% (*N* = 10), 28% (*N* = 12), 29% (*N* = 7), and 38% (*N* = 3), respectively (Supplementary File 2).

**Fig. 1:**
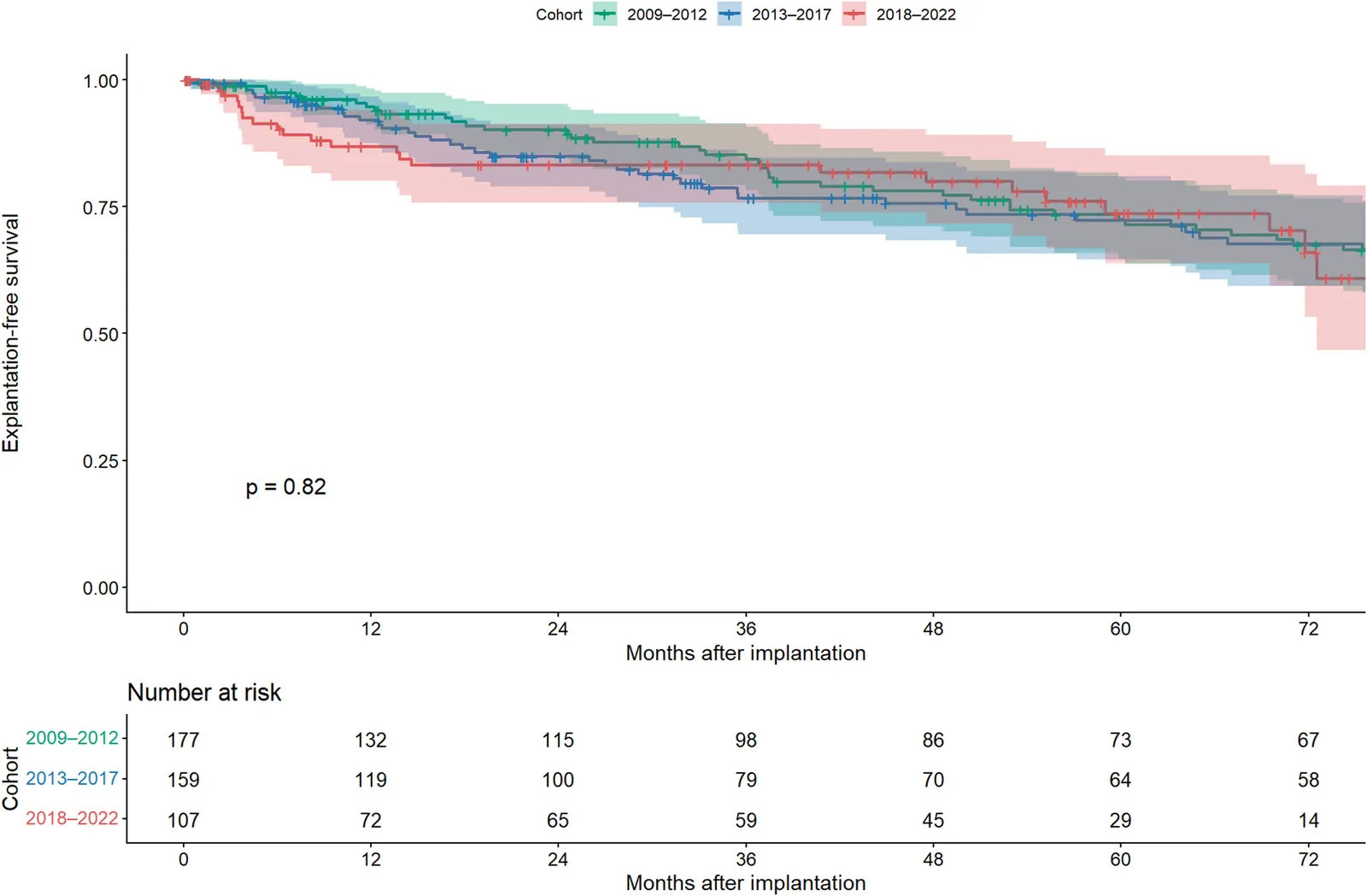
Kaplan–Meier curves for explantation-free survival stratified by implantation period

Kaplan-Meier analysis demonstrated no statistically significant differences (*p* = 0.8) in explantation-free survival between the time periods (Fig. 1), although explantation events appeared to occur earlier in more recent cohorts compared with earlier implantation periods.

### Risk factors for explantation

To identify risk factors for explantation, univariable and multivariable Cox proportional hazards regression analyses were performed (Table 2).

**Table 2:** Univariable and multivariable Cox regression analysis of factors associated with explantation of the artificial urinary sphincter

Variable	Univariable, HR (95%CI)	*p*-value	Multivariable, HR (95%CI)	*p*-value
Age (years)				
Per 1-year increase	1.02 (1.00–1.05)	0.065	1.02 (0.99–1.05)	0.1
Hypertension				
Yes vs. No (ref.)	1.39 (0.94–2.07)	0.1	1.03 (0.60–1.79)	0.9
Hyperlipidaemia				
Yes vs. No (ref.)	1.61 (1.12–2.30)	0.01	1.40 (0.85–2.30)	0.2
Diabetes mellitus				
Yes vs. No (ref.)	1.41 (0.93–2.15)	0.1	1.29 (0.84–1.98)	0.3
Smoking				
Yes vs. No (ref.)	1.41 (0.93–2.13)	0.1	1.36 (0.89–2.10)	0.2
Prior radiotherapy				
Yes vs. No (ref.)	1.75 (1.25–2.45)	0.001	1.88 (1.30–2.70)	< 0.001
Prior BPH surgery				
Yes vs. No (ref.)	1.64 (1.08–2.49)	0.02	1.75 (1.10–2.77)	0.02
Prior male sling				
Yes vs. No (ref.)	0.85 (0.54–1.36)	0.5	0.95 (0.58–1.54)	0.8
Prior AUS				
Yes vs. No (ref.)	1.72 (0.90–3.27)	0.1	1.46 (0.71–2.97)	0.3
Prior DVIU				
Yes vs. No (ref.)	1.10 (0.76–1.60)	0.6	0.82 (0.54–1.24)	0.3
Prior bulbar urethroplasty				
Yes vs. No (ref.)	2.43 (1.41–4.18)	0.001	2.10 (1.12–3.93)	0.02
Prior complex urethral reconstruction				
Yes vs. No (ref.)	1.15 (0.65–2.04)	0.6	1.81 (0.96–3.41)	0.065

*AUS *Artificial Urinary Sphincter; *BPH* Benign Prostatic Hyperplasia; *CI * Confidence Interval; *DVIU*  Direct Vision Internal Urethrotomy; *HR * Hazard Ratio; ref. reference category

In multivariable analysis, prior radiotherapy (HR 1.88, 95% CI 1.30–2.70), prior BPH surgery (HR 1.75, 95% CI 1.10–2.77;), and prior bulbar urethroplasty (HR 2.10, 95% CI 1.12–3.93) were independently associated with an increased risk of explantation. Age, hypertension, hyperlipidaemia, diabetes mellitus, smoking status, prior male sling implantation, prior AUS implantation, and prior DVIU were not independently associated with explantation in the multivariable model.

In the sensitivity analysis including implantation period, prior radiotherapy (HR 1.78, 95% CI 1.22–2.60), prior BPH surgery (HR 1.69, 95% CI 1.04–2.73), and prior bulbar urethroplasty (HR 2.39, 95% CI 1.24–4.60) remained significant predictors of explantation (Supplementary File 3). In contrast, implantation period was not significantly associated with explantation risk (2013–2017 vs. 2009–2012: HR 1.02, *p* = 0.9; 2018–2022 vs. 2009–2012: HR 0.97, *p* = 0.9).

### Supplementary analysis: early explantation outcomes

A supplementary analysis was performed focusing on early explantation within the first 24 months after AUS implantation. Early explantation rates within the first 24 months were 7.9% (*N* = 14) in 2009–2012, 13% (*N* = 20) in 2013–2017, and 14% (*N* = 15) in 2018–2022. In this early failure subgroup, urethral erosion and infection-related complications, either alone or in combination, represented the predominant causes of explantation across all cohorts (Supplementary File 4).

Kaplan-Meier analysis restricted to the first 24 months demonstrated no statistically significant difference (*p* = 0.2; Supplementary File 5) in early explantation rates between implantation periods, although higher rates were observed in more recent cohorts. To further investigate risk factors for early explantation, a multivariable Cox regression model was performed including established risk factors associated with a fragile urethra (Supplementary File 6). Prior radiotherapy (HR 2.46, 95% CI 1.38–4.39) and prior urethral surgery (HR 2.50, 95% CI 1.31–4.77) were independently associated with an increased risk of early explantation. Later implantation periods were associated with higher early explantation risk (2013–2017 vs. 2009–2012: HR 1.50, *p* = 0.2; 2018–2022 vs. 2009–2012: HR 1.71, *p* = 0.2), although this did not reach level of statistical significance.

## Discussion

In this single-center study, baseline patient characteristics and risk factors remained largely stable over time, although the prevalence of prior radiotherapy was higher in more recent cohorts. Explantation-free survival was comparable across implantation periods, although more recent cohorts showed a numerically higher proportion of early explantations. Prior radiotherapy, previous BPH surgery, and prior urethral surgery were identified as independent predictors of explantation.

The evolving profile of patients undergoing AUS implantation observed in this study reflects broader trends in the surgical management of male SUI. Contemporary patients increasingly present with complex treatment histories, including prior pelvic radiotherapy, repeated endoscopic interventions, and reconstructive urethral surgery [12, 13]. Although the relative increase in prior radiotherapy across implantation periods in our cohort did not reach statistical significance, likely reflecting the limited sample size of the most recent cohort, this numerical trend is in line with previously reported patterns of increasing patient complexity among contemporary AUS candidates and should be regarded as a descriptive clinical observation rather than a statistically confirmed finding. This development aligns with the growing recognition of the fragile urethra as a clinically relevant entity, characterized by impaired tissue quality and reduced vascularization, which increases vulnerability to complications such as erosion and infection [11, 14]. This concept is further supported by our findings, as erosion and infection represented the most common reasons for device explantation.

Despite increasing patient complexity, overall explantation outcomes remained stable across implantation periods. This observation may be explained by advances in surgical decision-making and technique, as well as higher institutional volume, surgical expertise, and standardized perioperative protocols [15]. Risk-adapted approaches, including alternative cuff placement strategies such as distal and transcorporal implantation in selected high-risk patients, may help mitigate adverse baseline characteristics [16–18]. In addition, increasing institutional experience and the centralization of complex reconstructive procedures are likely to contribute to improved perioperative management and complication handling, as suggested by recent multicenter data [4].

Notably, although overall explantation-free survival remained stable, a numerical increase in early explantations within 24 months was observed in more recent cohorts, suggesting a shift toward earlier failure patterns. These early events were predominantly driven by infection- and erosion-related complications, which are characteristic of the early postoperative phase and likely reflect underlying tissue vulnerability. To further explore early failure patterns, a supplementary Cox proportional hazards analysis was performed, adjusting for established risk factors of urethral vulnerability (fragile urethra) and implantation period. In line with previous reports, prior pelvic radiotherapy and prior urethroplasty were independently associated with earlier explantation, whereas prior incontinence surgery did not reach statistical significance [11]. Although implantation period itself was not independently associated with early explantation in multivariable analysis, the observed clustering of early failures in more recent cohorts may reflect shifts in baseline patient risk profiles over time, including a higher prevalence of patients with a fragile urethra [19]. In contrast, patients without such risk factors tend to demonstrate longer device survivorship, with later explantations more often attributable to mechanical failure or progressive loss of continence efficacy.

Prior radiotherapy, prior BPH surgery, and prior urethral surgery were identified as independent risk factors for explantation, underscoring their relevance in preoperative risk stratification and likely reflecting cumulative urethral damage with impaired tissue vascularization, thereby increasing the risk of erosion and infection [20, 21]. Implantation period itself was not independently associated with overall explantation risk in multivariable analysis. However, cuff placement strategy evolved over the study period, with an increasing proportion of distal double-cuff, and particularly transcorporal cuff placements in more recent cohorts, reflecting both a growing awareness of the fragile urethra concept and an increasing proportion of complex, previously treated patients. As transcorporal cuff placement is reserved for the most compromised urethral conditions, often as a salvage procedure, this shift in cuff selection may itself represent a marker of increasing patient complexity and could explain the numerically higher rate of early explantations observed in recent cohorts, rather than reflecting a decline in surgical quality or perioperative management.

With the increasing prevalence of risk factors and more complex patient profiles undergoing AUS implantation, individualized risk assessment is essential to align patient expectations with realistic outcomes. Although overall device durability appears preserved, clinicians should be aware of a higher likelihood of early complications in high-risk individuals and adapt follow-up strategies accordingly. Enhanced preoperative counselling may be particularly important in patients with a history of radiotherapy or prior urethral surgery. This temporal pattern underscores the potential value of defining an intermediate clinical endpoint based on standardized reporting of complication timing and etiology [22], which may help distinguish early biologically driven failures from later mechanically driven device failure and improve both follow-up strategies and patient counselling.

Several limitations of this study should be considered. First, this is a single-center study conducted at a high-volume reconstructive referral center with substantial expertise in the surgical management of male SUI, which may limit generalizability. As a tertiary referral institution, a higher proportion of complex cases, including patients with prior urethral interventions, pelvic radiotherapy, and compromised urethral integrity, is likely managed, introducing potential referral-related selection bias. In addition, cuff type was assigned based on institutional risk-adapted protocol, and the current analysis was not stratified by cuff placement; this decision-based selection may have influenced explantation outcomes and limits direct comparability with centers using different cuff selection strategies. Given the limited statistical power in later cohorts, temporal trends in comorbidities and urethral risk factors should be interpreted with caution and as hypothesis-generating rather than confirmatory. The retrospective design inherently carries a risk of selection and information bias. Although data were collected systematically, variations in follow-up duration and assessment intensity over time may have influenced outcome detection, and shorter follow-up in the most recent cohort may underestimate long-term complication rates. In addition, subgroup analyses may be underpowered to detect smaller effect sizes. To mitigate time-related bias, survival analyses were restricted to comparable follow-up periods where appropriate; however, residual bias cannot be fully excluded.

## Conclusion

The AUS remains a durable and effective treatment option for male SUI, with preserved overall outcomes across different implantation periods. However, an increase toward earlier explantation in more recent patients may indicate subtle shifts in contemporary failure patterns, potentially related to changes in patient risk profiles and early postoperative complication dynamics. Preoperative counselling, particularly in high-risk patients with prior radiotherapy or urethral surgery, remains essential to ensure realistic expectation setting and informed decision-making. Close postoperative follow-up in contemporary AUS candidates may facilitate early identification and management of complications to optimize overall treatment outcomes.

## Supplementary Information

Below is the link to the electronic supplementary material.

Supplementary Material 1

## Data Availability

The data that support the findings of this study are not publicly available due to patient privacy and data protection regulations. Anonymized data may be made available upon reasonable request to the corresponding author.
