# Supplementary material for: Temporal trends in patients undergoing artificial urinary sphincter implantation: explantation outcomes and early failure patterns in a tertiary reconstructive referral center
Source: World J Urol. 2026 Nov 13;44(1):802. doi: 10.1007/s00345-026-06899-9 (PMC13768383; doi:10.1007/s00345-026-06899-9)
Supplement: Supplementary file 1 [file 345_2026_6899_MOESM1_ESM.docx]

Supplementary File 1:

Table: Artificial urinary sphincter cuff placement across implantation periods

| Variable | 2009-2012;  N=177 | 2013-2017;  N=159 | 2018-2022;  N=107 | 2023-2025;  N=48 | p-value |
| --- | --- | --- | --- | --- | --- |
| Bulbar single cuff, N (%) | | | | | 0.9 |
|  | 49 (28) | 38 (24) | 26 (24) | 12 (25) |  |
| Distal double cuff, N (%) | | | | | 0.3 |
|  | 117 (66) | 114 (72) | 65 (61) | 31 (65) |  |
| Transcorporal cuff, N (%) | | | | | <0.001 |
|  | 6 (3.4) | 4 (2.5) | 15 (14) | 5 (10) |  |
| Other, N (%) | | | | | 0.6 |
|  | 5 (4.3) | 3 (1.9) | 1 (0.9) | 0 (0) |  |

Supplementary File 2:

Table: Causes of artificial urinary sphincter explantation across implantation periods

| Variable | 2009-2012;  N=63 | 2013-2017;  N=43 | 2018-2022;  N=24 | 2023-2025;  N=8 | p-value |
| --- | --- | --- | --- | --- | --- |
| Erosion, N (%) | | | | | 0.8 |
|  | 20 (32) | 13 (30) | 6 (25) | 2 (25) |  |
| Infection, N (%) | | | | | 0.035 |
|  | 3 (4.8) | 8 (19) | 4 (17) | 2 (25) |  |
| Erosion + Infection, N (%) | | | | | 0.3 |
|  | 10 (16) | 12 (28) | 7 (29) | 3 (38) |  |
| Mechanical failure, N (%) | | | | | 0.6 |
|  | 2 (3.2) | 2 (4.7) | 1 (4.2) | 0 (0) |  |
| Loss of function, N (%) | | | | | 0.02 |
|  | 17 (27) | 5 (12) | 2 (8.3) | 0 (0) |  |
| Other / Combinations, N (%) | | | | | 0.6 |
|  | 11 (17) | 3 (6.9) | 4 (17) | 1 (13) |  |

Supplementary File 3:

Table: Sensitivity analysis of risk factors for artificial urinary sphincter explantation including implantation periods (multivariable Cox regression analysis)

| Variable | Multivariable, HR (95%CI) | p-value |
| --- | --- | --- |
| Age (years) | | |
| Per 1-year increase | 1.02 (0.99-1.05) | 0.2 |
| Hypertension | | |
| Yes vs. No (ref.) | 1.05 (0.59-1.87) | 0.9 |
| Hyperlipidaemia | | |
| Yes vs. No (ref.) | 1.48 (0.87-2.52) | 0.5 |
| Diabetes mellitus | | |
| Yes vs. No (ref.) | 1.23 (0.78-1.93) | 0.4 |
| Smoking | | |
| Yes vs. No (ref.) | 1.20 (0.76-1.92) | 0.4 |
| Prior radiotherapy | | |
| Yes vs. No (ref.) | 1.78 (1.22-2.60) | 0.003 |
| Prior BPH surgery | | |
| Yes vs. No (ref.) | 1.69 (1.04-2.73) | 0.033 |
| Prior male sling | | |
| Yes vs. No (ref.) | 0.98 (0.60-1.61) | 0.9 |
| Prior AUS | | |
| Yes vs. No (ref.) | 1.55 (0.74 – 3.25) | 0.2 |
| Prior DVIU | | |
| Yes vs. No (ref.) | 0.80 (0.52-1.23) | 0.3 |
| Prior bulbar urethroplasty | | |
| Yes vs. No (ref.) | 2.39 (1.24-4.60) | 0.009 |
| Prior complex urethral reconstruction | | |
| Yes vs. No (ref.) | 1.99 (1.05-3.78) | 0.036 |
| Period of implantation | | |
| 2013-2017 vs. 2009-2012 (ref.) | 1.02 (0.67-1.54) | 0.9 |
| 2018-2022 vs. 2009-2012 (ref.) | 0.97 (0.57-1.64) | 0.9 |

*AUS = Artificial Urinary Sphincter; BPH = Benign Prostatic Hyperplasia; CI = Confidence Interval; DVIU = Direct Vision Internal Urethrotomy; HR = Hazard Ratio; ref. = reference category*

Supplementary File 4:

Table: Causes of early artificial urinary sphincter explantation within 24 months across implantation periods

| Variable | 2009-2012;  N=14 | 2013-2017;  N=20 | 2018-2022;  N=15 | p-value |
| --- | --- | --- | --- | --- |
| Erosion, N (%) | | | | 0.5 |
|  | 3 (21) | 5 (25) | 6 (40) |  |
| Infection, N (%) |  |  |  | 0.4 |
|  | 1 (7.1) | 5 (25) | 3 (20) |  |
| Erosion + Infection, N (%) | | | | 0.9 |
|  | 4 (29) | 7 (35) | 5 (33) |  |
| Mechanical failure, N (%) | | | | 0.08 |
|  | 2 (14) | 0 (0) | 0 (0) |  |
| Loss of function, N (%) | | | | 0.029 |
|  | 4 (29) | 2 (10) | 0 (0) |  |
| Other / Combinations, N (%) | | | | 0.9 |
|  | 0 (0) | 1 (5) | 1 (6.7) |  |

Supplementary File 5:

Figure: Kaplan-Meier analysis of explantation-free survival by implantation period restricted to the first 24 months

Supplementary File 6:

Table: Sensitivity analysis of risk factors (fragile urethra) for artificial urinary sphincter explantation within 24 months including implantation periods

| Variable | Multivariable, HR (95%CI) | p-value |
| --- | --- | --- |
| Prior radiotherapy | | |
| Yes vs. No (ref.) | 2.46 (1.38-4.39) | 0.002 |
| Prior urethral surgery | | |
| Yes vs. No (ref.) | 2.50 (1.31-4.77) | 0.005 |
| Prior incontinence surgery | | |
| Yes vs. No (ref.) | 0.96 (0.45-2.05) | 0.9 |
| Period of implantation | | |
| 2013-2017 vs. 2009-2012 (ref.) | 1.50 (0.76-2.98) | 0.2 |
| 2018-2022 vs. 2009-2012 (ref.) | 1.71 (0.82-3.55) | 0.2 |

*CI = Confidence Interval; HR = Hazard Ratio; ref. = reference category*
